# DNA vaccines encoding the envelope protein of West Nile virus lineages 1 or 2 administered intramuscularly, via electroporation and with recombinant virus protein induce partial protection in large falcons (*Falco* spp.)

**DOI:** 10.1186/s13567-015-0220-1

**Published:** 2015-08-17

**Authors:** Dominik Fischer, Joke Angenvoort, Ute Ziegler, Christine Fast, Kristina Maier, Stefan Chabierski, Martin Eiden, Sebastian Ulbert, Martin H. Groschup, Michael Lierz

**Affiliations:** Clinic for Birds, Reptiles, Amphibians and Fish, Justus Liebig University Giessen, Frankfurter Str. 91-93, 35392 Giessen, Germany; Friedrich-Loeffler-Institut, Federal Research Institute for Animal Health, Institute of Novel and Emerging Infectious Diseases, Südufer 10, 17493 Greifswald-Insel Riems, Germany; Fraunhofer Institute for Cell Therapy and Immunology, Perlickstraße 1, 04103 Leipzig, Germany

## Abstract

As West Nile virus (WNV) can cause lethal diseases in raptors, a vaccination prophylaxis of free-living and captive populations is desirable. In the absence of vaccines approved for birds, equine vaccines have been used in falcons, but full protection against WNV infection was not achieved. Therefore, two DNA vaccines encoding the ectodomain of the envelope protein of WNV lineages 1 and 2, respectively, were evaluated in 28 large falcons. Four different vaccination protocols were used, including electroporation and booster-injections of recombinant WNV domain III protein, before challenge with the live WNV lineage 1 strain NY99. Drug safety, plasmid shedding and antibody production were monitored during the vaccination period. Serological, virological, histological, immunohistochemical and molecular biological investigations were performed during the challenge trials. Antibody response following vaccination was low overall and lasted for a maximum of three weeks. Plasmid shedding was not detected at any time. Viremia, mortality and levels, but not duration, of oral virus shedding were reduced in all of the groups during the challenge trial compared to the non-vaccinated control group. Likewise, clinical scoring, levels of cloacal virus shedding and viral load in organs were significantly reduced in three vaccination groups. Histopathological findings associated with WNV infections (meningo-encephalitis, myocarditis, and arteritis) were present in all groups, but immunohistochemical detection of the viral antigen was reduced. In conclusion, the vaccines can be used safely in falcons to reduce mortality and clinical signs and to lower the risk of virus transmission due to decreased levels of virus shedding and viremia, but full protection was not achieved in all groups.

## Introduction

West Nile virus (WNV) is a arthropod-borne *Flavivirus* belonging to the *Flaviviridae* family and the Japanese encephalitis serogroup complex [1]. At least seven different lineages of WNV have been demonstrated by phylogenetic analysis [2,3], with lineages 1 and 2 being of high zoonotic importance [4]. The virus is distributed worldwide, except for Antarctica [1,5] and epidemics in birds caused by linage 1 and 2 have been reported from different regions in Europe [6-8].

Following an enzootic life cycle, WNV is transmitted between arthropods, especially some mosquito species, and a wide range of vertebrates [9,10]. Birds are regarded as important virus reservoirs [11], whereas humans and mammals (especially horses) represent mainly dead-end hosts, potentially suffering from febrile disease, encephalitis, meningitis, poliomyelitis, and death [4,12,13]. In the latter, viremia may be low, but in reptilian and avian species, viral titers have been demonstrated to be high enough for re-infection of mosquitoes [14]. Therefore, migratory birds play an important role in spreading WNV [15-17].

Besides asymptomatic courses, WNV infections may lead to severe morbidity and mortality in different avian species, especially in raptors, crows and domestic geese [18-27], which seemed to be particularly vulnerable to WNV. In raptors, natural WNV infections have been described in hawks [20,21,28-30], eagles [24-26,31-33], condors [34] and different falcon species, such as peregrine falcons (*Falco peregrinus*), gyrfalcons (*F. rusticolus*), prairie falcons (*F. mexicanus*), merlins (*F. columbarius*) and American kestrels (*F. sparverius*) [27,30]. Experimental WNV infection studies have been performed in single raptor species [27,35-37], demonstrating that falcons can act as amplifying hosts, developing viremia, virus shedding, and subclinical to fatal diseases.

Although WNV constitutes an obvious threat to free-ranging and captive birds of prey in public or private collections, zoos, falconries or commercial breeding centers, a vaccine destined for birds is not available. However, inactivated vaccines and subunit vaccines destined for horses, as well as experimental DNA vaccines, have been used in avian species and evaluated either by the development of neutralizing antibodies [34,38,39] or by experimental WNV challenge [40-51]. Inactivated WNV vaccines and recombinant canary pox vaccines showed limited efficacy in large falcons, especially if administered only twice before an experimental WNV lineage 1 challenge infection [51]. Eventually, a triple application scheme of both vaccines (given at three-weekly-intervals) reduced the clinical signs sufficiently, decreased or even abolished WN-viral RNA shedding and prevented residual viral organ loads three weeks post-challenge. However, full protection against a clinical WNV infection was not always achieved.

DNA WNV vaccines have been used with very promising results in crows [40,41], songbirds [45], and raptors [34,46]. In American robins (*Turdus migratorius*), American crows (*Corvus brachyrhynchos*) and fish crows (*Corvus ossifragus*), viremia levels were effectively reduced by the intramuscular administration of DNA vaccines. Additionally, in crows, higher survival rates in WNV challenge trials were achieved by vaccination compared to controls. California condors (*Gymnogyps californianus*) showed neutralizing antibody titers sixty days after vaccination, which have been considered as effective protection against naturally circulating WNV during the 2004 transmission season [34]. In red-tailed hawks (*Buteo jamaicensis*), the stimulation of antibody development following twofold vaccination was very low (only 21.4% of the vaccinated birds had low titers), but a significant reduction in mean viremia levels was achieved after challenge [46]. However, differences in seroconversion rate and virus shedding were not significant and the absence of morbidity and mortality in the control group during WNV challenge trial limits its validity for clinical protection.

In light of the observed drawbacks of other vaccination strategies in large falcons, we tested a DNA vaccine candidate in combination with different application schemes and confirmed its efficacy by live virus challenge. The vaccine encodes the envelope (E) protein of WNV, the major target for neutralizing antibodies [52]. Our results indicate that protective immune responses are induced upon DNA vaccination. However, the level of protection is strongly dependent on the delivery system, with in-vivo electroporation (EP) being the most successful.

## Materials and methods

### Animals and viruses

In total, 28 captive-bred, mature (>6 month old) falcons (*Falco rusticolus* [*n* = 6]; *F. cherrug* [*n* = 1]; *F. peregrinus* [*n* = 3]; *F. cherrug x F. rusticolus* [*n* = 14]; *F. peregrinus x F. rusticolus* [*n* = 6]) originating from one collection were used for this study. All birds were healthy in clinical and endoscopic examination and were dewormed twice (3 week interval) prior to the trial (25 mg/kg fenbendazol orally, Panacur Suspension 2.5% ad us. vet., MSD Animal Health GmbH, Unterschleißheim, Germany and 25 mg/kg toltrazuril orally, Baycox 5% orale Suspension ad us. vet., Bayer Animal Health GmbH, Leverkusen, Germany). ELISA (ID Screen© WN competition ELISA, IDVet, Grabels, France) and the micro-virus neutralization test (VNT) confirmed the absence of pre-existing WNV specific antibodies. Additionally, the absence of Usutu virus specific antibodies was confirmed by VNT using the USUV strain Vienna_2001 (GenBank accession no. AY453411.1).

WNV lineage 1 NY99 (GenBank accession no. AF196835) was propagated and titrated on Vero E6 cells and used for virus challenge according to previous studies [37,51]. Virus identity was verified by sequencing the WNV E protein gene. Challenge virus doses contained 10^6^ Tissue Culture Infection Dose 50 (TCID_50_) diluted in 1 mL minimal essential medium (MEM) and were injected subcutaneously in the inguinal region.

### Construction of WNV DNA vaccine candidates

Two DNA vaccines containing a modified version of the pVax1 vector (Invitrogen, Karlsruhe, Germany) as the backbone were prepared according to Schneeweiss et al. [52].

The first vaccine (WNV-DNA-1) encoded the E protein ectodomain of WNV lineage 1 strains WNV 2000-crow3356 (GenBank accession no. AF404756.1), which is a close relative to the challenge WNV NY99 strain.

The second vaccine (WNV-DNA-2) encoded the E protein ectodomain of WNV lineage 2 with the sequence of the E protein of WNV strain goshawk Austria 361/10 (2009) (GenBank accession no. HM015884). DNA for intramuscular application was dissolved in 0.9% (w/v) sodium chloride.

All DNA‐preparations used for vaccination in this study were checked for their ability to express the E‐protein via transient transfection assays in cultured cells, as described in Schneeweiss et al. [52] before being injected into animals (data not shown).

### Expression and purification of recombinant WNV proteins

The recombinant domain III of WNV 2000-crow3356 was generated as a fusion protein with the maltose binding protein (MBP), as described by Schneeweiss et al. [52]. The MBP tag was enzymatically removed from domain III by factor X cleavage according to the manufacturers’ instructions (New England Biolabs, USA). Here, 250 μg of protein in 150 μL phosphate buffered saline (PBS) was mixed with 350 μL adjuvant Montanide ISA 70 VG (Seppic, France) (= 500 μL in total) and used for vaccination.

### Falcon immunizations and WNV challenge studies

During the time of vaccination, falcons were housed in outdoor hexagonal aviaries with a ground area of 125 m^2^ and height of 6 m in a commercial breeding center. The area of the center was inspected and approved by the animal welfare (permission no.: 392016/2009) and species conservation authorities (permission no.: AZ 68.337-02/4.2) and approved as an area for genetic engineering (BS003115275-1 dt Az. 40611/5801/501) according to the German Genetic Engineering Act (Gentechnikgesetz, GenTG). Vaccination studies were approved (reference no. 7221.3-1.1.-056/10) on the basis of the EU council directive 86/609/EEC for the protection of animals used for experiments. Vaccines were administered under isoflurane inhalation anesthesia [53].

Each of the four vaccination groups (A-D) consisted of five randomly selected falcons plus two randomly selected, unvaccinated control falcons (which were merged into a control group E (*n* = 8) eventually).

**Figure 1:**
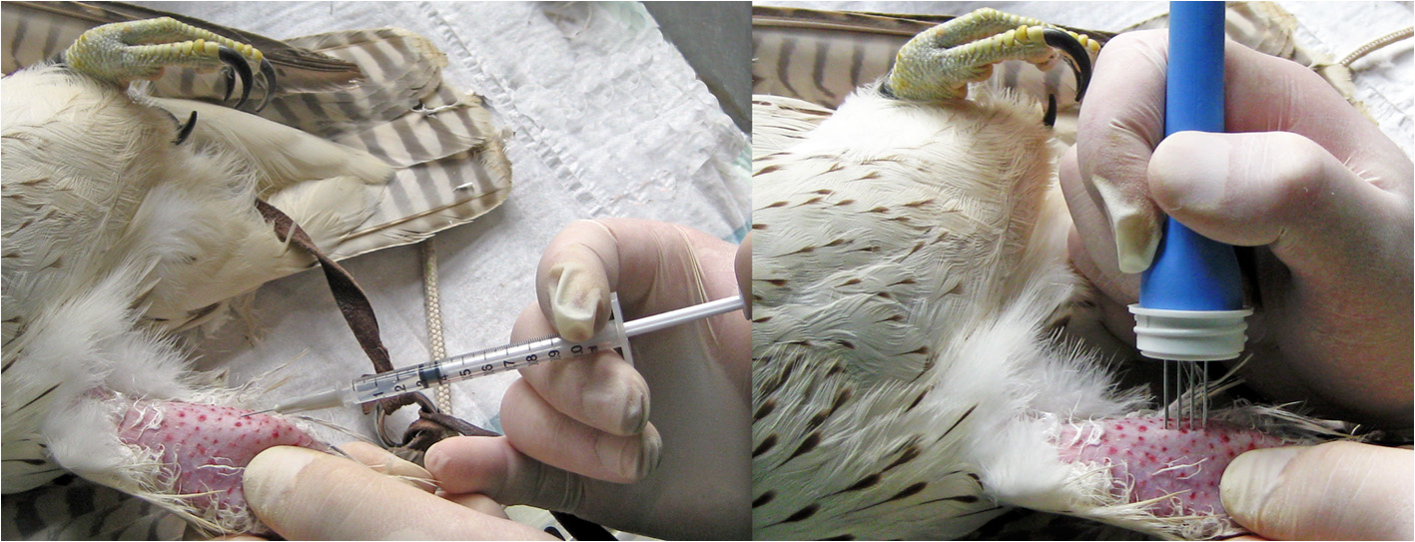
**Vaccination and application of in-vivo electroporation in falcons.** The intramuscular injection of the vaccine and the application of in-vivo electroporation using a four-electrode array and an electric pulse generator (Clinivet, IGEA, Modena, Italy) are shown on right lower leg muscles of a falcon in group C. The feathers were plucked in a 3 cm^2^ area around the injection site.

Falcons in groups A and B were vaccinated by intramuscular injection (pectoral muscle) with a plasmid (700 μg per dose) encoding the E protein of the WNV lineage 1 (group A) or 2 (group B) and immune responses were boosted once after three weeks post-vaccination (wpv). Falcons in groups C and D received a plasmid (75 μg per dose) encoding WNV E protein lineage 1 by intramuscular injection into the lower leg muscles immediately followed by an in-vivo electroporation (EP) using the electric pulse generator Clinivet (IGEA, Modena, Italy) according to Schneeweiss et al. [52]. For this purpose, feathers were plucked, exposing an area of 3 cm^2^ around the injection site, and the four-electrode array was placed on the skin to apply the electric pulses (Figure 1). While group C animals were boosted after three weeks in the same way, falcons of group D received two subcutaneous injections of recombinant WNV E protein domain III and ISA 70 adjuvant after two and four weeks. Depending on the assigned vaccination group, the control animals (group E) received injections of sodium chloride, EP and/or adjuvant, respectively, at the same time as the respective vaccinated birds. Each bird was kept under constant observation for 3 h after each treatment. During the seven weeks of the vaccination course, the health status of all falcons was assessed daily and a clinical examination was performed every seven days, including palpation of the vent, auscultation of the lungs and heart, and inspection of nares, pharynx, mucus membranes, feet, skin and muscles at the vaccination sites. Blood samples and swabs from the oropharynx and cloaca were taken weekly for WNV antibody determination by ELISA, VNT [51] and DNA plasmid detection by PCR, respectively.

After seven (groups A & B) or six weeks (groups C & D), respectively, falcons were transferred to an animal trial unit and challenged under biosafety level 3 conditions with WNV strain 1 NY99. Challenged falcons were examined daily for general condition, posture, plumage, behavior, excrements, neurological status, hydration status, respiration and food uptake [51]. Measurements of body temperature, weight, condition score and hydration status were performed under isoflurane inhalation anesthesia, and samples were taken (blood, pharyngeal and cloacal swabs) on 0, 3, 6, 8, 10, 12, 14 and 19/20/21 days post-infection (dpi) [51]. Clinical findings were documented using a clinical scoring system: a score of 0 referred to a healthy/inconspicuous status, 1 to a mildly affected status, score 2 to a moderately affected status and 3 to a severely affected status. Deceased animals were given a score of 4. Birds were euthanized on 19/20/21 dpi or earlier, when falcons showed seizures, torticollis, somnolence or apathy. Post-mortem examinations were performed on all dead birds, and included the sampling of organs (lung, heart, spleen, kidney, liver, and brain) and subsequent storage of samples in supplemented MEM at −70 °C and in formalin, as described earlier [37,51].

### Antibody detection

Sera were assayed by ID Screen© WN competition ELISA (IDVet) following the manufacturer’s instructions, with a 40% competition as a cut-off value. Additionally, VNT on Vero cells were conducted using two-fold dilutions (in MEM) and homologous challenge virus (100 TCID_50_) following the protocol by Seidowski et al. [54]. Cytopathogenic effects were assessed after seven days using formalin-fixation and crystal violet staining, and neutralizing titers were calculated using the Behrens-Kaerber method, as previously described [51]. Similarly, sera were checked for neutralizing antibodies against USUV using the strain Vienna_2001 (GenBank accession no. AY453411.1) for VNT.

### Detection of plasmid shedding

During the vaccination period, pharyngeal and cloacal swabs were examined for the presence of WNV plasmid DNA originating from WNV-DNA-1 and WNV-DNA-2. For this purpose, swabs were incubated in PBS and DNAs were extracted using E.Z.N.A Plasmid Mini Kit I (Omega bio-tek, Norcross, GA, USA). Afterwards, 10 μL eluate was examined by PCR using Taq polymerase (NEB, Ipswich, MA, USA) in a Biometra Thermocycler and four different primers (WPN-L1-fwd: 5′-CCTCCCTTGGAGCAGTGCTGGA-3′; WPN-L1-rev: 5′-CCTCCCTTGGAGCAGTGCTGGA-3′; WPN-L2-fwd 5′-TTGTGCATGGCCCGACGACT-3′; WPN-L2-rev: 5′-CCGGCCAGAGCTTGGTGCAAT-3′) with 394 and 379 base pairs, respectively. A detection level of 0.5 pg plasmid was determined per PCR.

### Virus isolation and viral genome detection

Virus isolation was performed according to Angenvoort et al. [51] using serial dilutions of organ homogenate supernatants and bovine serum albumin-1 (BA-1) dilutions for the inoculation of Vero cells. After seven days, cells were formalin-fixed and stained with crystal violet; virus titers were calculated using the Spearman and Kaerber method, as described recently [51]. Viral RNAs were isolated from oral and cloacal swabs, from organ homogenate supernatants using the QIAamp® Viral RNA Kit (Qiagen) and from blood in BA-1 using the RNeasy® Mini Kit (Qiagen) according to the manufacturer’s instructions [51]. In parallel with these samples, an internal control RNA (IC RNA) containing 2 × 10^5^ copies/μL was extracted. Viral RNA and IC RNA were stored at −70 °C for further analysis. Two qRT-PCRs, amplifying the 5′NTR-region or the NS2A-region, respectively, were used for WNV virus detection and quantification as described before [55]. Cycle threshold (Ct) values below 35 were regarded as positive, from 35 to 40 as suspicious and above 40 as negative.

### Histological and immunohistochemical examinations

Formalin-fixed tissue samples were embedded in paraffin, cut into 3 μm sections, rehydrated and stained with hematoxylin/eosin (H&E) or by using monoclonal antibodies (mabs) to WNV in immunohistochemistry (IHC). Mab 15R4 (kindly provided by P. Emmerich, Bernhard Nocht Institut, Hamburg, Germany) and mab 3B2 (kindly provided by Davide Lelli, Istituto Zooprofilattico Sperimentale della Lombardia e dell’Emilia Romagna, Brescia, Italy), were diluted in goat serum (1:20 and 1:80, respectively) and the slides were immunostained as described before [51]. Briefly, endogenous peroxidase was blocked using H_2_O_2_/methanol for 30 min. As a pretreatment, a Proteinase K digestion for 15 min at 37 °C using a concentration of 4 μg/mL (mab 15R4) and heating with citrate buffer (pH 6.0) for 10 min (mab 3B2) were applied, respectively. The slides were finally developed using Mouse Envision HRP (Dako Diagnostics, Dako Deutschland GmbH, Hamburg, Germany) and diaminobenzidine. Hearts, spleens, brains and the virus injection sites were evaluated and scored for their percentage of positive tissues/cells according to previous studies [51]. Thereby, an organ score of 0 was negative (= no cells affected), 1 was mildly affected (<1% positive cells), 2 was moderately affected (≥1% and <5% positive cells) and 3 was severely affected (≥5% positive cells).

### Statistical analysis

WNV antibodies were compared within and between birds from vaccination and control groups using Fisher’s exact test. The mean duration of illness and clinical scores of all days for each falcon were compared using one-sided Wilcoxon rank sum test with continuity correction. All time points were calculated separately, except 19, 20 and 21 dpi, which were averaged to one terminal time point. For testing the duration of illness, a clinical score of 4 was excluded from the analysis. Each group was compared to the control group separately, whereby *p*-values below α-levels of 0.05 were classified as significant.

Levels of oral and cloacal virus shedding and of viremia were analyzed using ANOVA including all sampling days. The duration of virus shedding and viremia were evaluated using one-sided Wilcoxon rank sum test with continuity correction and a cut-off Ct value of 35.0. Likewise, this test was used for the analysis of viral organ loads (in heart, spleen, kidney, brain). All statistical tests were performed using R software [56].

## Results

The efficacy of two different WNV DNA vaccines in falcons was determined by a) assessing their tolerance, safety and potency to elicit specific antibody responses and b) by challenging the vaccinated and control birds with a semi-lethal WNV NY99 dose.

### Vaccine tolerance and safety

Following vaccination and sampling, behavior, food uptake, movement or general health of the falcons were not affected at any time. The body weight of the vaccinated and control animals varied only slightly between +3.5% and −6.6%. In group B, two birds (F32, F33) developed an edematous/gelatinous texture of pectoral muscle 1 wpv. One falcon in group D demonstrated 0.4 cm × 0.5 cm sized, yellowish, dense skin texture at the vaccination site 1 and 2 weeks after boost vaccination (F56, 5-6 wpv) with recombinant WNV protein. The vaccination sites of all other birds were macroscopically unsuspicious. In none of the oral and fecal swabs from the falcons was WNV plasmid DNA detected at any time.

### Vaccination induced antibody responses

**Figure 2:**
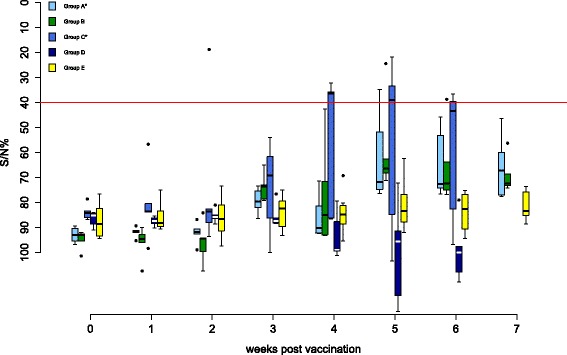
**Antibodies detected by ID Screen© WN competition ELISA during the vaccination period in falcons.** Antibodies of all groups detected by the ID Screen© WN competition ELISA (IDVet, Grabels, France) during the vaccination period are shown. Optical density at 450 nm is converted to signal/noise% (S/N%) ratio (S/N% = ODsample/ODnegative control * 100), with values ≤40% considered positive, >40% and ≤50% being equivocal and >50% negative. The threshold for positive results is indicated as a solid red line. In box-and-whisker plots, the ends of the whiskers represent the minimum and maximum values, respectively. Outliers are represented as black dots instead of whisker-ends and the box includes 50% of the values for each group. Vaccination groups with a statistically significant difference in development of antibody production detected by ELISA and micro-virus neutralization test in comparison to group E are labeled with an asterisk (Fisher’s exact test, *p*-values < α-levels of 0.05).

**Figure 3:**
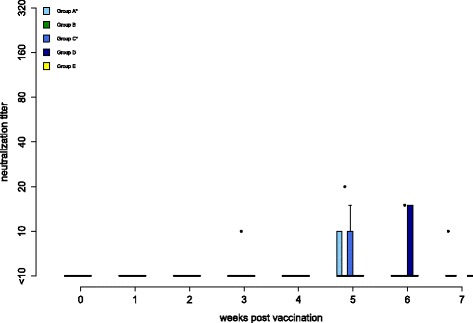
**Antibodies detected by the micro-virus neutralization test (VNT) against homologous challenge WNV.** Antibody titers of all groups determined by VNT against the homologous challenge virus (neutralization titer) during the vaccination period are shown. In box-and-whisker plots, the ends of the whiskers represent the minimum and maximum values, respectively. Outliers are represented as black dots instead of whisker-ends and the box includes 50% of the values for each group. Vaccination groups with statistical significant difference in development of antibody production detected by ELISA and micro-virus neutralization test in comparison to group E are labeled with an asterisk (Fisher’s exact test, *p*-values < α-levels of 0.05).

Antibody responses are shown in Figures 2 and 3. All control birds (group E) remained negative by ELISA and VNT. In group A, one bird (F25) was ELISA-positive from 5 wpv onwards. At the same time, two other falcons (F29, F30) had low antibody titers of 1:10 by VNT, which lasted for 3 weeks in one bird. All other individuals of this group remained negative in both tests throughout the complete trial.

In group B, one falcon (F31) was ELISA-positive 4 wpv and another falcon (F33) had a low antibody titer of 1:20 5 wpv by VNT. Both individuals were negative at all other time points examined and all of the other birds of this group remained negative throughout the entire vaccination period.

Three falcons in group C (F49, F52, F53) were positive by ELISA from 4 wpv onwards. One of these (F52) and one other falcon (F50) had antibody titers of 1:15 and 1:10 by VNT, respectively, lasting for 1-2 weeks.

None of the birds in group D were positive by ELISA and only two birds (F57, F58) had an antibody titer of 1:15 by VNT 6 wpv.

In groups A and C, but not in groups B and D, Fisher’s exact test demonstrated a significantly increased presence of WNV antibodies compared to group E.

### Clinical signs following WNV NY99 challenge

**Figure 4:**
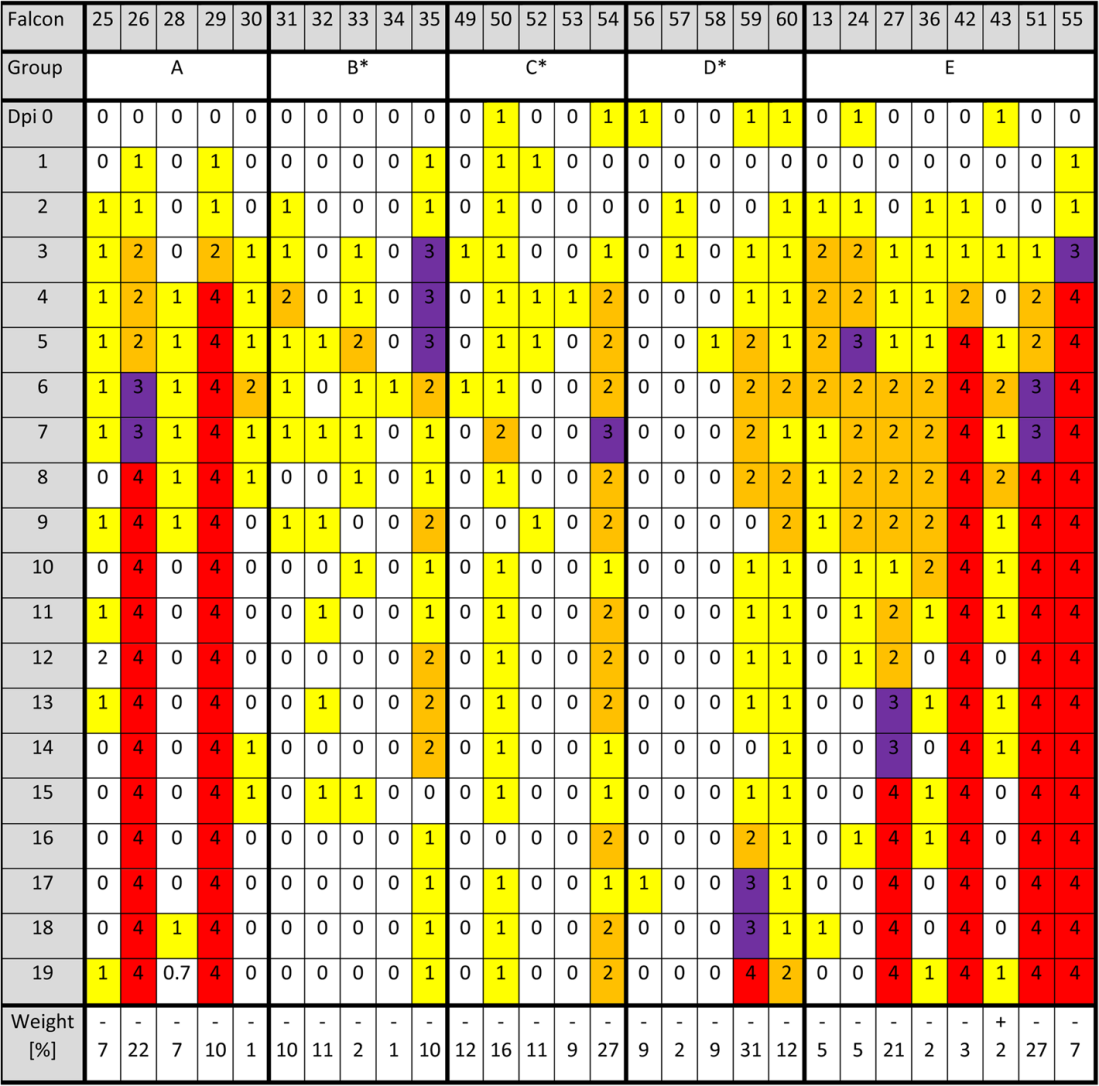
**Clinical score: following criteria were classified daily: general condition, posture, plumage, behavior, excrements, neurological status, hydration status, respiration and food uptake (alterations: +++ = 3 points, ++ = 2 points, + = 1 point, +/ - = 0.5 points and - = 0 points).** Points were summed up and total points of one day were classified in a clinical score system: 0–0.5 points = 0 = healthy; 1–4.5 points = 1 = mild affection; 5–10.5 points = 2 = moderate affection; 11 points and more = 3 = severe affection; 4 = dead. Weight presents the change of body weight (− = decrease, + = increase) in percent as comparison of the first and the last measurement of each falcon during the trial. Vaccination groups with statistical significant difference in sum of clinical scores in comparison to group E are labelled with an asterisk (one-sided Wilcoxon rank sum test with continuity correction, p-values < α-levels of 0.05). Abbreviation: dpi (days post infection).

Details on the clinical scores and development of body weights are provided in Figure 4. In the control group E, two of the eight control birds (F55, F27) had to be euthanized 3 dpi and 14 dpi, respectively, due to severe central nervous signs (clinical score 3) such as seizures, torticollis, ataxia and an inability to stand. Two other birds (F42, F51) were found dead on 5 dpi and 8 dpi, respectively, after showing greenish discoloration of urates, enophthalmos, inappetence and ruffled feathers on the previous day. Similar symptoms (clinical scores 2-3) were observed in the four other control birds between 3 dpi and 11 dpi temporarily, but clinical signs ceased afterwards. All control birds dropped their weight from 1.56% to 27.01% (median: 5.75%).

Four falcons of group A had clinical scores equal to or above 2 and two falcons (F26, F29) died on 4 dpi and 8 dpi, respectively. Body weight dropped slightly in the three survivor birds (0.7-7.1%) and decreased rapidly and severely in the two deceased falcons (21.9% and 10.5%, respectively). Median weight loss of all birds in group A was 6.8%.

In group B, two falcons (F31, F33) had clinical scores of 2 on one day and one falcon (F35) had clinical scores of 2 and 3 over several days. No bird died and weight losses ranged from 0.76% to 10.9% (median: 9.6%).

One falcon (F50) of group C had a clinical score of 2 on one day (7 dpi) and another bird (F54) had this score after ten days. However, clinical score in the latter increased to 3 for one day. No falcon died and the weight loss in four birds ranged from 9.1% to 16% (median: 11.6%), whereas in one bird (F54), it was up to 27.1%.

In group D, one falcon (F60) had a clinical score of 2 and one falcon (F59) had a clinical score of 3. The latter animal was euthanized on 18 dpi because of ataxia, hypermetria and weight loss of 30.9%. The other birds in this group dropped weight from 1.5% to 12% (median: 8.7%).

In groups B, C and D, but not in group A, significantly reduced clinical scores were demonstrated by Wilcoxon rank sum test compared to the control group E (*p*-values 0.01154, 0.02391, 0.04592; for group A 0.1703).

### Virus shedding, viremia and antibody response after challenge infection

**Figure 5:**
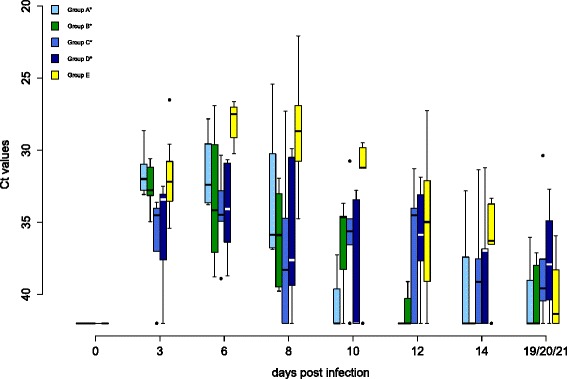
**Oral virus shedding of falcons during WNV lineage 1 challenge.** The oral virus shedding of falcons during challenge is displayed. Cycle threshold values (Ct values) of swabs for all groups are shown. In box-and-whisker plots the ends of the whiskers represent the minimum and maximum values, respectively. Outliers are represented as black dots instead of whisker-ends. This includes 50% of the values of each group, with the median value of each group being represented by a line in the middle. Vaccination groups with statistical significant difference in the level of oral viral genome shedding in comparison to group E are labeled with an asterisk (ANOVA over all sampling days, *p*-values < α-levels of 0.05). There was no group with a statistically significant difference in duration of oral viral genome shedding (one-sided Wilcoxon rank sum test with continuity correction, cut-off Ct value 35.0, *p*-values < α-levels of 0.05).

**Figure 6:**
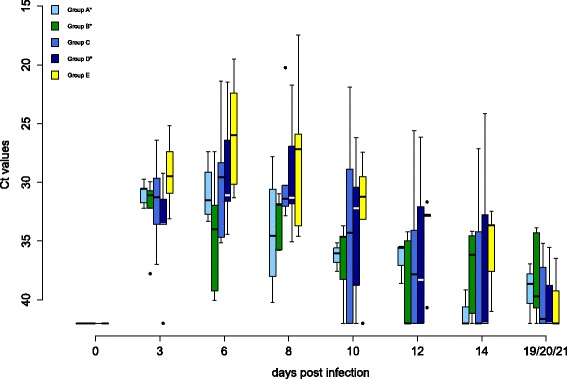
**Cloacal virus shedding of falcons during WNV lineage 1 challenge.** The cloacal virus shedding of falcons during challenge is displayed. Cycle threshold values (Ct values) of swabs for all groups are shown. In box-and-whisker plots the ends of the whiskers represent the minimum and maximum values, respectively. Outliers are represented as black dots instead of whisker-ends. The box includes 50% of the values of each group and median value of each group is represented by a line in the middle. Vaccination groups with a statistically significant difference in the level of cloacal viral genome shedding in comparison to group E are labeled with an asterisk (ANOVA over all sampling days, *p*-values < α-levels of 0.05). There was no group with a statistically significant difference in duration of cloacal viral genome shedding (one-sided Wilcoxon rank sum test with continuity correction, cut-off Ct value 35.0, *p*-values < α-levels of 0.05).

**Figure 7:**
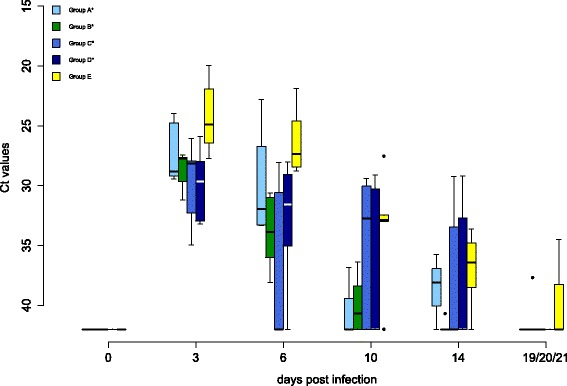
**Viremia of falcons during WNV lineage 1 challenge detected by qRT-PCR.** The cycle threshold values (Ct values) of whole blood for all groups during the challenge period are displayed. In box-and-whisker plots the ends of the whiskers represent the minimum and maximum values, respectively. Outliers are represented as black dots instead of whisker-ends. The box includes 50% of the values of each group and median viremia value of each group is represented by a line in the middle. Vaccination groups with a statistically significant difference in the level of viremia in comparison to group E are labeled with an asterisk (ANOVA over all sampling days, *p*-values < α-levels of 0.05). There was no group with a statistically significant difference in duration of viremia (one-sided Wilcoxon rank sum test with continuity correction, cut-off Ct value 35.0, *p*-values < α-levels of 0.05).

**Figure 8:**
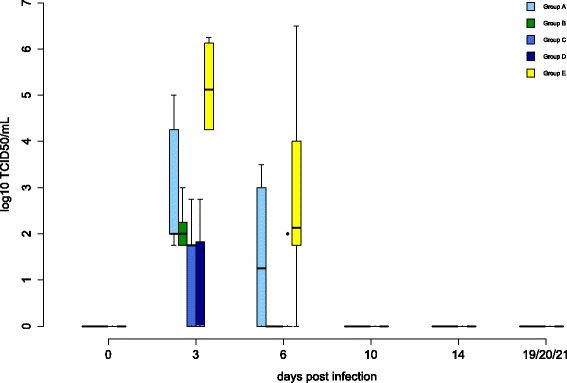
**Viremia of falcons during WNV lineage 1 challenge detected by virus titration.** The results of blood titration for all groups during the challenge period are given in log10 tissue culture infection dose 50 per mL (TCID50/mL) whole blood. In box-and-whisker plots the ends of the whiskers represent the minimum and maximum values, respectively. Outliers are represented as black dots instead of whisker-ends. The box includes 50% of the values of each group and the median viremia value of each group is represented by a line in the middle.

**Figure 9:**
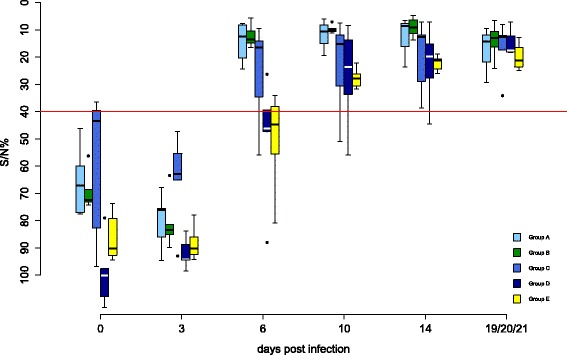
**Antibodies detected by ELISA during challenge in falcons.** Antibodies of all groups detected by the ID Screen© WN competition ELISA (IDVet, Grabels, France) during the challenge period are shown. Optical density at 450 nm is converted to signal/noise% (S/N%) ratio (S/N% = ODsample/ODnegative control * 100), with values ≤ 40% considered positive, >40% and ≤50% equivocal and >50% negative. The threshold for positive results is indicated as a solid red line. In box-and-whisker plots, the ends of the whiskers represent the minimum and maximum values, respectively. Outliers are represented as black dots instead of whisker-ends and the box includes 50% of the values for each group.

**Figure 10:**
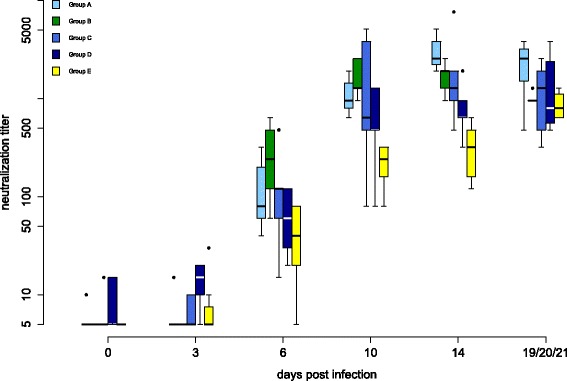
**Antibodies detected by micro-virus neutralization test (VNT) during challenge in falcons.** Antibody titers of all groups determined by VNT against homologous challenge virus (neutralization titer) during the challenge period are shown. In box-and-whisker plots the ends of the whiskers represent the minimum and maximum values, respectively. Outliers are represented as black dots instead of whisker-ends. The box includes 50% of the values of each group and median value of each group is represented by a line in the middle.

Oral and cloacal virus shedding data are shown in Figures 5 and 6, virus genome detection in the blood in Figure 7 and virus re-isolation data from blood in Figure 8. Serology results are depicted in Figures 9 and 10.

All birds of the challenge control group (group E) shed WNV orally and in their feces from 3 dpi onwards, with Ct values varying from 17.48 to 34.75. However, in the four surviving control birds (F13, F24, F36, F43), viral genomes were sometimes not detected (F36 at 20 dpi and F13, F24, F43 after 8-14 dpi). Viral genomes were detected in the blood of all falcons from 3 dpi onwards, with Ct values ranging from 19.94 to 34.78. Viremia ceased in most of the survivors on 6-14 dpi (all except F36), while it persisted in the fatally affected falcons until death. Virus was successfully re-isolated and titrated from blood taken on 3 and 6 dpi (yielding titers in the range of 10^1.75^-10^6.5^ TCID 50/mL respectively). First, WNV-specific neutralizing antibodies were detected on 3 dpi (F36, F55) or 6 dpi (F13, F24, F27, F43), respectively, and ranged from 1:10 to 1:80. However, two of the eight falcons which did not survive the WNV challenge did not develop a neutralizing antibody response (F42, F51). In the survivors (F13, F24, F36, F43), the antibody titers increased to a maximum of 1:1280 at the end of the observation period.

In oral and cloacal swabs and in blood taken from birds of group A, viral genomes were only detectable between 3 dpi and 8 dpi. A solitary positive result in one bird (F28, Ct value of 32.82 at 14 dpi), which was negative by qRT-PCR at all other time points, may be due to an artifact. Virus re-isolations from blood were successful in all birds on 3 dpi (10^1.75^-10^5^ TCID 50/mL) and in two birds (F25, F26) also from samples taken at 6 dpi (10^2.5^ and 10^3.5^ TCID50/mL). Antibody titers from 1:15 to 1:320 were detectable in one bird (F30) from 3 dpi and in three birds from 6 dpi onwards, increasing to a maximum of 1:5120 during the trial. However, in the two birds from this group which eventually died, antibody titers remained low (1:40) after challenge (F26) or disappeared (F29) after being low (1:10) before challenge.

In group B, animal viral genomes were detected in pharyngeal swabs up to 10 dpi and in cloacal swabs of two birds (F34, F35) even until 19-21 dpi. Birds were viremic on 3 and 6 dpi, but not at later time points. Virus titers in blood on 3 dpi were 10^1.75^-10^3^ TCID50/mL. Initial antibody titers on 6 dpi ranged from 1:60 to 1:640 and rose to maximum titers of 1:2560 at the end of the study.

In group C, viral genomes were detected in oral and pharyngeal swabs and in blood up to 14 dpi. One bird (F54) shed virus orally until 21 dpi. Blood Ct values were in the range of 21.40 to 34.5. Virus re-isolations were successful from blood samples taken from three birds on 3 dpi (titers 10^1.75^-10^2.75^ TCID50/mL), but not from blood samples taken at later time points. Antibody titers were detected in two falcons (F52 and F53) already by 3 dpi and in all falcons on 6 dpi. One of these falcons (F52) carried already antibodies on 0 dpi as a result of the vaccination. Initially ranging from 1:10 to 1:480, antibody titers increased to maximum titers 1:7680 and lasted until to the end of the study.

In group D, viral genomes were detected in pharyngeal swabs beginning at 3 dpi until the end of the observation period and until 14 dpi in cloacal swabs and blood. Virus re-isolations were successful in two birds (F60, F59) on 3 dpi with 10^1.83^ TCID50/mL and 10^2.75^ TCID50/mL, respectively, and additionally in one of these birds on 6 dpi (10^2^ TCID50/mL). Antibody titers in four birds ranged from 1:10 to 1:20 on 3 dpi; however, antibodies were present on 0 dpi in two of these birds (F57, F58). The titers increased in all birds, up to maximum levels of 1:3840. One bird (F59) with a low neutralizing antibody titer on 6 dpi (1:20), but without a strong humoral response (maximum titers of 1:320), showed neurological signs and apathy and was eventually euthanized on 18 dpi.

ANOVA demonstrated that oral (*p* values groups A-D: 0.0006594, 0.000002827, 0.00006856, 0.000122) and cloacal (*p* values groups A, B, D: 0.0005033, 0.0002035, 0.03737; for group C 0.06771) virus shedding and virus detection in blood (*p* values groups A-D: 0.001057, 0.000000268, 0.0006269, 0.0005634) were significantly reduced in all groups compared to the control group. However, the duration of oral shedding (*p* values groups A-D: 0.1013, 0.2041, 0.4702, 0.3014) and viremia (*p* values groups A-D: 0.1566, 0.09665, 0.4108, 0.4108) was not significantly different, as determined by the Wilcoxon rank sum test.

### Post mortem examination, histopathology and immunohistochemistry

A summary of the pathological and immunohistochemical findings is provided in Table 1.

**Table 1:** **Histopathological (HE) and immunohistochemical (IHC) results from vaccinated falcons and non-vaccinated controls infected with WNV lineage 1 NY’99**

Grp	ID	Dpi	Brain		Heart		Spleen	Inject.	Vacc.		Others								
			HE	IHC	HE	IHC	IHC	IHC	Qual	Deg	Art	PALS	Neu	Nep	Hep	Ent	Pan	Ser	Sep
A	F25	20	2	0	1	0	0	1	-	0	1	0	0	0	0	1 c	0	0	0
	F26	8*	2	1	0	1	0	1	-	0	0	3	0	0	0	0	0	0	0
	F28	21	2	0	1	0	0	0	-	0	0	1	1	1	0	0	0	0	0
	F29	4*	0	0	0	0	0	1	-	0	0	2	0	0	0	3 h	0	0	0
	F30	19	2	1	0	0	0	0	-	0	1	1	1	0	0	0	0	0	0
B	F31	21	2	0	0	0	0	0	Nsp.	1	1	0	0	0	1	0	0	0	0
	F32	19	2	0	1	0	0	0	-	0	0	0	0	0	0	0	0	1	0
	F33	21	1	0	1	0	0	0	-	0	0	0	0	0	0	1 c	0	0	0
	F34	20	1.5	0	0	0	0	0	-	0	0	0	1	0	0	0	0	0	0
	F35	19	0	0	0	0	0	0	-	0	0	0	0	0	0	0	1	0	0
C	F49	20	2.5	0	0	0	0	0	Nsp.	0.5	1	0	1	0	0	0	0	0	0
	F50	20	3	0	0.5	0	0	0	-	0	0	0	1	0	0	0	0	0	0
	F52	19	0.5	0	0	0	0	0	Fol.		0	0	0	0	0	1 c	0	0	0
	F53	19	0	0	0	0	1	0	Fol.		2	0	0	0	0	0	0	0	0
	F54	21	2	1	1	1	0	0	-	0	0	0	1	1	0	0	0	0	0
D	F56	19	1	0	0	0	0	0	-	0	0	0	1	0	1	1 c	0	0	0
	F57	21	2.5	0	0	0	0	0	Fol.		0	0	0	0	0	0	0	0	0
	F58	21	2	0	10	0	0	0	Gran.	3	0	0	1	1	0	0	0	0	0
	F59	18*	2	3	1.5	0	0	0	Nsp.	1	0	0	1	0	0	0	0	0	0
	F60	20	1.5	0	1.5	0	0	0	Gran.	2	1	0	1	0	0	0	0	0	0
E	F13	19	2	0	0.5	0	0	0	ND		0	0	1	0	0	0	0	0	0
	F24	20	2	1	2	0	0	0	ND		2	0	1	0	1	0	1.5	1	0
	F27	14*	1.5	1	3	0	0	1	ND		0	0	1	1.5	0	1	0	0	0
	F36	20	1.5	0	1	0	0	0	ND		0	0	1	0	0	0	0	0	0
	F42	5*	1	0	0	0 p	1	2	ND		0	3	1	0	0	0	0	0	0
	F43	20	1.5	0	2	1	1	1	ND		0	0	1	0	0	1	1.5	0	0
	F51	8*	0.5	0	0	1	3	2	ND		0	3	0	1.5 u	1	0	0	0	0
	F55	3*	0	0	0	1	0	3	ND		0	3	0	0	0	0	0	0	2

Unspecific findings seen in most of the 28 challenged animals included a pale (26/28) and enlarged spleen (24/28) with severe lymphoid depletion (20/28) and a marked lymphcytolysis in the remaining follicles (20/28), bloated foamy macrophages in spleen (23/28) and in liver (27/28) which shows also an extramedullary hematopoiesis (24/28).

Abbreviations: Grp (group); ID (identification number); Dpi (days post infection); * (died/euthanized before end of the trial); Inject. (virus injection site); Vacc. (vaccination site); HE (hematoxylin and eosin stain); IHC (immunohistochemistry); Qual (quality); Deg (degree); Art (arteriitis); PALS (periarteriolar lymphoid sheat); Neu (neuritis); Nep (nephritis); Hep (hepatitis); Ent (enteritis); Pan (pancreatitis); Ser (serositis); Sep (septicemia); Nsp. (non-suppurative inflammation); Fol. (follicular aggregated mononuclear cells); Gran. (granulomatous inflammation); p (petechia); u (uratnephritis); c (detection of coccidial parasites); h (hemorrhagic).

The numbers for HE examination indicate a weak (0.5), mild (1), mild to moderate (1.5), moderate (2), moderate to severe (2.5) and severe (3) alteration. The score for IHC indicate no positive cells (0), ≤1% positive (1), >1% and ≤5% positive (2) and >5% positive tissue structures (3).

The gross-pathology, histopathology and IHC of the non-vaccinated positive controls (group E) were previously described [51]. Briefly, the most consistent finding associated with WNV infection was a non-suppurative (meningo)-encephalitis (4/8) and an acute or subacute, lymphohistiocytic, necrotizing myocarditis (5/8). A moderate, non-suppurative, necrotizing arteritis was seen in the spleen of one bird only (F24). Virus antigens were detected by IHC in brains, hearts and spleens and at injection sites of the fatally diseased animals (4/8), whereas challenge survivors displayed only scant (2/8) or no (2/8) WNV antigen staining.

**Figure 11:**
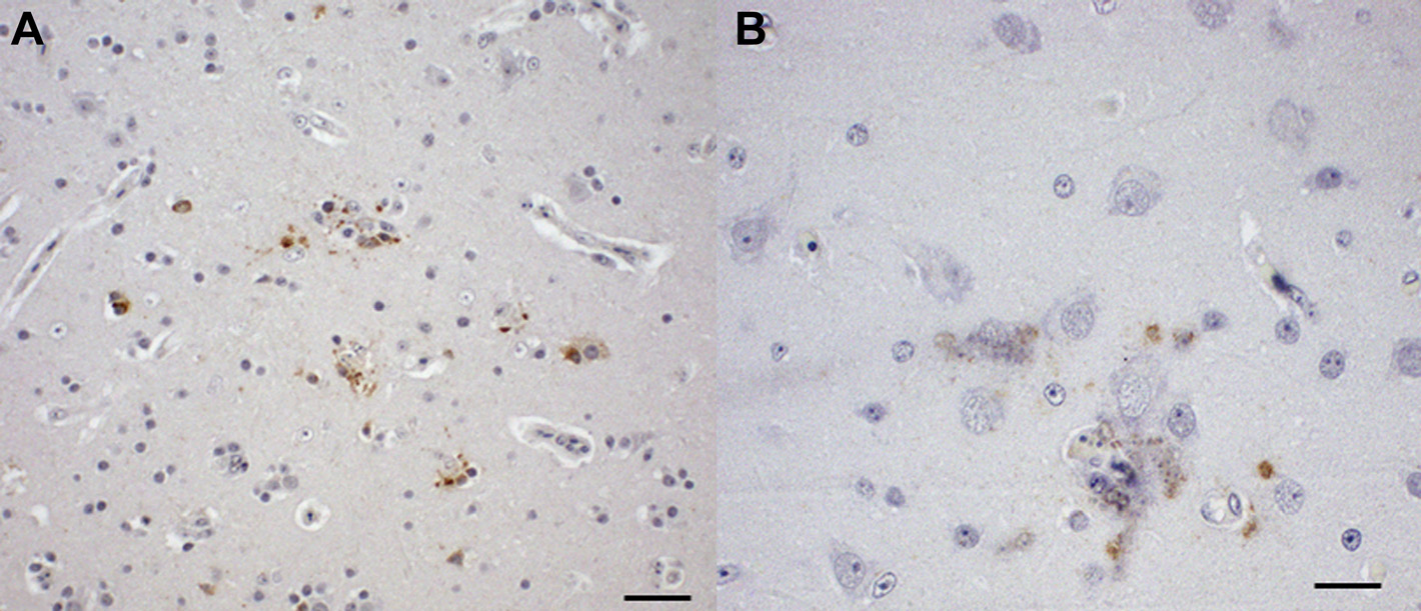
**Immunohistochemistry of the cerebrum of vaccinated falcons.** Single cell necrosis and mild glial reaction with distinct amounts of viral antigen within neurons and glial cells are demonstrated in the cerebra of two falcons. **(A)** Falcon in group D, 18 dpi, mab 15R4, bar 50 μm. **(B)** Falcon in group A,19 dpi, mab 15R4, bar 20 μm.

One falcon (F29) of group A died 4 dpi and showed a severe, acute, hemorrhagic enteritis with positive antigen staining at the injection site, but without other signs typically associated with WNV infections. In all other birds (4/5) a widely distributed, moderate, acute, non-suppurative (meningo)-encephalitis was diagnosed. Additional findings included an acute to subacute, mild, necrotizing myocarditis (2/5), an acute, mild, necrotizing arteritis in the kidney (2/5), a necrosis of the splenic periarteriolar lymphoid sheaths (PALS) of different degrees with fibrin deposition (4/5), a mild lymphoplasmacellular interstitial nephritis (1/5) and a mild lymphohistiocytic neuritis with axonal degeneration (2/5). The vaccination sites were not altered in any bird of this group. By IHC, scattered antigen was detected at the virus injection sites in three birds (3/5) and in the brains of two birds (2/5) (Figure 11), with one of these (F 26) having antigen in all regions of the brain and additionally in the heart.

One falcon in group B (F35) demonstrated only a slight lymphohistiocytic pancreatitis. All other animals (4/5) demonstrated a mild to moderate, widely distributed, acute, non-suppurative meningoencephalitis. Additionally, a mild, acute, necrotizing myocarditis (2/5), a mild, necrotizing arteritis in the gut connective tissue (1/5), a mild lymphohistiocytic hepatitis (1/5) and neuritis (1/5) were seen. The vaccination site of one animal (1/5) displayed a mild lymphohistiocytic myositis. No WNV antigen was detected by IHC in any bird of this group.

**Figure 12:**
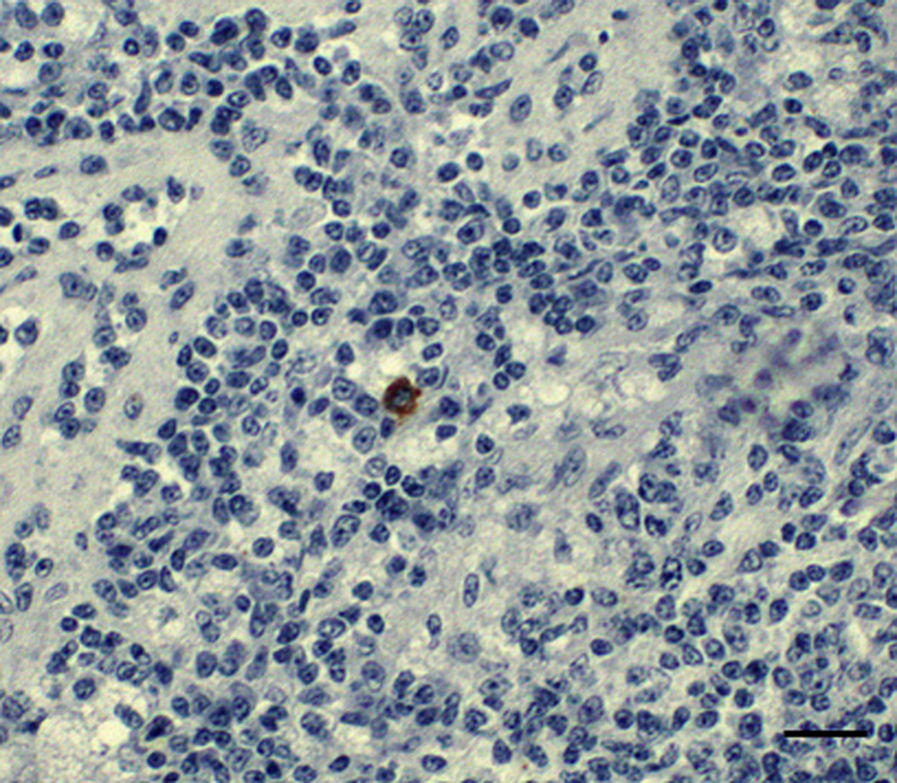
**Immunohistochemistry of the spleen of a vaccinated falcon.** The spleen of a falcon in group C, 19 dpi is displayed. Antigen was detected in single mononuclear cells in follicles, mab 15R4, bar 50 μm.

Four birds (4/5) of group C had acute, non-suppurative encephalitis of different degrees, varying from slight lesions (1/4) in the cerebellum/cerebrum to moderate and severe inflammation of all brain areas (1/4) and the meninges (2/4). Additional findings included an acute necrotizing myocarditis (2/5), a mild to moderate, acute, necrotizing arteritis (2/5), a mild lymphohistiocytic neuritis (3/5) and nephritis (1/5). At the vaccination sites, follicular aggregated infiltrations of mononuclear cells with mild lymphocytolysis (2/5) and an acute lymphohistiocytic myositis (1/5) were seen. IHC revealed a mild staining reaction in the spleen or brain and heart, respectively, of two falcons (2/5) (Figure 12), whereas all other birds remained negative.

**Figure 13:**
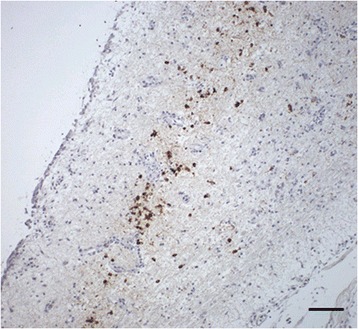
**Immunohistochemistry of the telencephalon of a vaccinated falcon.** The telencephalon of a falcon in group D, 18 dpi, is displayed. Regions close to ventricles, most notably the area parahippocampalis, were severely affected, mab 15R4, bar 100 μm.

An acute, non-suppurative meningoencephalitis of varying degrees was observed in all birds from group D (5/5). Additional findings included an acute to subacute, mild to moderate, necrotizing myocarditis (3/5), a mild, acute, necrotizing arteritis (1/5), and a mild, acute to subacute, non-suppurative neuritis (4/5) and nephritis (1/5). The vaccination sites had infiltrations of follicular aggregated mononuclear cells in the connective tissues (1/5) and a granulomatous dermatitis (2/5). In IHC, one bird (F59, Figures 11 and 13) was severely affected in cerebrum (15% positive tissue) and mildly affected in cerebellum and diencephalon. However, IHC results in the spleen and brain of two falcons (F58, F60) were inconclusive.

In general, the occurrence of scattered antigen in neurons, glial cells and cell processes of the brain, in cardiocytes and in single monocytes and fibrocytes at the injection site of vaccinated falcons were similar to findings in the control birds.

### Virus loads in organs

The results of qRT-PCR and virology from selected organ samples are summarized in Table 2. In the organs of all falcons of group E viral genomes were detected with lowest Ct values of 16.7 (equivalent to 402 895 copies/mg). All organs of the four fatally affected birds (F27, F42, F51, F55) were qRT-PCR positive, as were 2-5 organs of the four challenge survivors (F13, F24, F36, F43). Accordingly, virus re-isolations were possible from organs of the four deceased birds in most cases (10^1.75^-10^4.75^ TCID50/100 μL). In the four survivors virus isolations were successful only from the brain samples of two birds (F13, F43).

**Table 2:** **Viral load of organs in Ct values, copies per mg organ and tissue culture infectious dose 50 (TCID50) per mg organ**

Falcons			Brain			Spleen			Kidney			Lung			Liver			Heart		
Grp	ID	Dpi	Ct	Copies	TCID	Ct	Copies	TCID	Ct	Copies	TCID	Ct	Copies	TCID	Ct	Copies	TCID	Ct	Copies	TCID
A	F25	20	30.5	40.6	0.5	N	0	0	N	0	0	N	0	0	N	0	0	N	0	0
	F26	8	17.4	360248.4	48899.3	18.4	287861	432	22.1	28394.8	558.7	29.1	384.8	1814	19.8	43699.3	312.8	21.1	43844.8	139.7
	F28	21	31.1	84.6	0	N	0	0	31	155.6	0	N	0	0	N	0	0	N	0	0
	F29	4	32.9	12.2	0.6	25.2	3941.3	26.8	26.5	1081.2	1.9	28.3	511.6	5.3	27.9	448.2	0.7	30.6	69	0
	F30	19	29.1	313.2	1	N	0	0	33.1	19.2	0	N	0	0	N	0	0	N	0	0
B	F31	21	33.1	3.3*	0	N	0*	0	N	0*	0	N	0	0	N	0	0	N	0*	0
	F32	19	33.1	2.6*	0	N	0*	0	32.6	6.2*	0	N	0	0	N	0	0	N	0*	0
	F33	21	N	0*	0	N	0*	0	N	0*	0	N	0	0	N	0	0	N	0*	0
	F34	20	29.4	38*	0	N	0*	0	N	0*	0	N	0	0	N	0	0	N	0*	0
	F35	19	N	0*	0	N	0*	0	N	0*	0	N	0	0	N	0	0	N	0*	0
C	F49	20	32.3	26.5	0	N	0*	0	N	0*	0	N	0	0	N	0	0	N	0*	0
	F50	20	31.4	36.3	0	N	0*	0	N	0*	0	N	0	0	N	0	0	N	0*	0
	F52	19	N	0	0	N	0*	0	N	0*	0	N	0	0	N	0	0	N	0*	0
	F53	19	N	0	0	N	0*	0	N	0*	0	N	0	0	N	0	0	N	0*	0
	F54	21	30.4	23.3	0	N	0*	0	N	0*	0	N	0	0	N	0	0	N	0*	0
D	F56	19	30.5	35.3	0	N	0	0	N	0*	0	N	0	0	N	0	0	N	0	0
	F57	21	26.7	192.7	0	N	0	0	32.3	6.6*	0	N	0	0	N	0	0	N	0	0
	F58	21	31.6	14	0	N	0	0	N	0*	0	N	0	0	N	0	0	N	0	0
	F59	18	19.9	40297.1	62.2	30.6	44.7	0	34.3	1.9	0	33.7	1.9	0	33.8	4.3	0	31.8	14	0
	F60	20	30.4	39.6	0	36.4	2.6	0	35.5	1.4*	0	N	0	0	N	0	0	N	0	0
E	F13	19	28.3	111.6	0.6	30.8	17.9	0	32.7	3.9	0	39.7	0.1	0	36.3	0.3	0	N	0	0
	F24	20	31.6	32.1	0	N	0	0	36.1	821.8	0	N	0	0	N	0	0	39.3	4.7	0
	F27	14	21.3	132202.1	8.7	31.8	42	0	28.9	403.3	0	33.1	36.9	0	34.2	16.7	0	24.4	7793.8	37.9
	F36	20	31	23.6	0	N	0	0	34.9	2	0	N	0	0	N	0	0	N	0	0
	F42	5	27.3	514.8	47	20.7	318323	174.6	23.2	14400	76.2	25	5456.3	600.8	23.2	32118.9	94.4	24.8	6562.9	19
	F43	20	30.9	38.2	0.4	33.7	11.4	0	36.5	1.5	0	N	0	0	41.4	0.1	0	41.5	0.1	0
	F51	8	22.7	2667.5	737.9	22.5	8631	10.6	18.9	82090.5	2895.9	19.8	63844.1	7558.4	16.7	402895.4	3023.2	17.7	295310.6	4379.9
	F55	3	31	12.3	0	23.9	2375.9	3.4	25.7	795.6	3.9	26.4	464.9	13.2	26.7	248.5	0.9	27.7	379.8	5.4

Abbreviations: Grp (group), ID (identification number), Dpi (days post infection), Ct (Ct values), Copies (copies per mg organ), TCID (tissue culture infectious dose 50 per mg organ), N (no Ct value). Viral genome copies of brain, spleen, kidney and heart of all surviving birds of the vaccination groups were compared to the respective values of group E with one-sided Wilcoxon rank sum test with continuity correction and those with statistical significant difference were indicated with an asterisk (p-values < α-levels of 0.05 were classified as significant).

In all birds of group A, viral genomes were detected in the brain and also in the kidneys in four birds. Heart, spleen, liver and lung were negative in the three survivors, but positive in two falcons (F26, F29) which died during the study. In the latter two falcons, viruses could be isolated from all organs (except from heart in F29), but only from brain (10^0.75^-10^5.75^ KID50/100 μL) in two other falcons (F25, F30) and from none of the organs in one falcon (F28).

Brains of three falcons (F31, F32, F34) belonging to group B carried viral genomes and the kidney of one falcon (F32) was also qRT-PCR-positive. None of the other birds and none of the organs were positive by qRT-PCR and virus re-isolation failed in all birds.

In group C, qRT-PCR was positive in three birds (F49, F50, F54) in the brain, but not in other organs. Viral genomes were not detected in any other bird and the virology was negative in all birds for all organs.

Viral genomes were detected in the brain in all birds in group D, in one falcon (F57) additionally in the kidneys and in another falcon (F59) additionally in all other organs. In the latter falcon, which had to be euthanized during the trial, virus re-isolation was successful from the brain with a result of 10^2.75^ TCID50/100 μL, whereas it failed in all other birds of this group in all organs.

Wilcoxon rank sum test revealed that the viral genome detections was significantly reduced in all organs of group B animals (*p* values of brain, spleen, kidney and heart: 0.01767, 0.02228, 0.009749, 0.008443), in the spleen, kidney and heart of group C animals (*p* values 0.03582, 0.005709, 0.01536; for brain 0.1286), and in the kidneys of group D animals (*p* value 0.04762), but not in the spleen, heart and brain of group D birds (*p* values 0.1726, 0.1187, 0.7262). No significant reduction of viral genome detection in organs was present in group A (*p* values of brain, spleen, kidney, and heart: 0.5476, 0.6532, 0.5476, and 0.5, respectively).

## Discussion

WNV infections can lead to subclinical or severe diseases and mortality in falcons [27,30,35,37,51]. Free-ranging birds play a major role in the spread of WNV over large distances [15-17]. In order to protect raptors, vaccines destined for use in horses have recently been tested in falcons [51]. As these show only sub-optimal efficacy, more vaccine candidates were tested in the present study. For this purpose, WNV DNA vaccines were chosen because they had been used in raptors and other avian species with promising results [34,40,45,46]. However, differences in their efficacy were observed for several avian species [46]. Therefore, a species-specific efficacy study was initiated to determine the rates of clinical protection and the reduction of virus shedding, viral load and virus distribution in large falcons.

In contrast to the WNV DNA vaccine used in previous studies in raptors, which coded for prM and E glycoproteins of WNV [40], in the present study, two DNA vaccines encoding a secreted version of the E protein ectodomain of WNV lineage 1 [52] (WNV-DNA-1) and 2 (WNV-DNA-2), respectively, were used. Previously, DNA vaccines have been administered intramuscularly into the leg muscles of raptors [34,46]. This administration corresponds to findings in dogs, robins, and crows, where influences of the administration routes on humoral responses were demonstrated. Intramuscular administration induced stronger antibody responses compared to oral or intradermal applications, respectively [40,41,45,57]. However, the pectoral muscle is generally the preferred site for intramuscular drug administration in birds, due to the larger muscle mass and possible rapid drug elimination by a renal portal venous system from the leg muscles. Therefore, the pectoral muscles were preferred for intramuscular injection in the falcons of groups A and B. Moreover, WNV-DNA-1 was administered using EP in two groups (groups C and D) and boosted in the same way (group C) or with recombinant WNV protein subcutaneously (group D) according to successful studies in mice [52]. As EP has only been sparsely reported in avian species [58] and impairment of heart function by the electric impulses in the adjacent thoracic muscles could not be ruled out, vaccination and EP were applied to the lower leg muscles in these groups.

Overall, the safety and acceptance of both vaccines and the EP were good. There was no alteration in behavior, food uptake, movement or general health condition in any bird. In comparison to previous studies in falcons using a recombinant canarypox vectored WNV vaccine [51] no remarkable loss of body weight was observed in this study, but there was also local inflammation at the vaccination sites. Two birds in group B developed an edematous/gelatinous texture of pectoral muscle which disappeared after two weeks and could not be confirmed at necropsy. Moreover, mild, non-suppurative myositis and follicular aggregates were seen at the vaccination sites of single birds in groups B, C and D. Two falcons of group D developed a moderate to severe granulomatous dermatitis. Taken together, most alterations were confined to groups which were vaccinated twice and electroporated. Therefore, a reaction to the extended vaccination scheme was suspected, possibly caused by hypersensitivity reactions to the vaccine, the adjuvant or the recombinant protein.

A possible shedding of the WNV vaccine plasmid by immunized birds was investigated by taking cloacal and pharyngeal swabs from all falcons on a weekly basis (data not displayed). The fact that WNV DNA plasmid was not found in any of these swabs highlights the environmental safety of these DNA plasmid vaccines used.

Elicited antibody titers were low in all four vaccination groups and lasted for a maximum of 3 weeks in single individuals. Moreover, many vaccinated birds failed to produce detectable antibody levels, or titers were detectable on single days only but not repeatedly over weeks. These results contrast strongly with DNA vaccination reports in California and Andean condors, where all birds demonstrated increased neutralizing antibody levels in plaque-reduction test 4 wpv [34]. It also lags behind other DNA vaccination studies, where in 15/19 American crows and in 6/9 fish crows, neutralizing antibodies were demonstrated [40,41]. Moreover, antibody levels were low compared to previous studies in large falcons, using formalin-inactivated WNV vaccines and recombinant canarypox live WNV vaccines [51]. However, similar differences have been shown in four penguin species, where higher antibody levels were induced by an inactivated vaccine compared to a DNA vaccine [39]. Low antibody titers had also been reported in red tailed hawks, where only 3/14 birds developed low titers (maximum titers 1:10) 4 wpv following DNA vaccination [46]. Moreover, in 3/3 American robins, no detectable antibody levels have been measured 2 wpv [45]. Thereby, species-specific differences may play a role in humoral response, as previously discussed [46].

To solve the problem of low immunogenicity of DNA vaccines, strategies such as in-vivo EP have been developed which result in a significant increase in humoral and cellular immune responses [59]. However, antibody responses were not markedly stronger using this approach in the present study. A likely explanation is that DNA vaccination in falcons mainly enhances cellular and/or innate immune mechanisms, as suggested for other species [39,41,52,58,60,61] This might explain the higher survival rates, the lower oral virus shedding and the lower viral organ load in groups C and D compared to group A.

Besides EP, antibody levels were recently further stimulated using recombinant protein DIII in mice [52]. However, this failed in falcons of group D compared to falcons of groups A and C in this study. This might be explained by a possible lower efficacy of the adjuvant ISA 70 in falcons compared to oligonucleotides (ODN 1826), which have been used in the mice [52]*.* The importance of the adjuvant has been shown in geese, where ISA720 induced significantly higher antibody titers compared to vaccination of the subunit protein antigen without this adjuvant [43]. In contrast, in crows, the adjuvant did not significantly affect the performance of the DNA vaccine [41].

Antibody responses to virus challenge were poor in the deceased or euthanized birds but were good in the surviving control birds. In the vaccinated birds, titers raised from 3 dpi onwards in group B, and even higher in EP groups C and D. Therefore, the first antibodies in vaccinated falcons were demonstrated three days earlier compared to the first antibody detection in 4/8 control birds and unvaccinated large falcons from a previous WNV infection trial [37]. In a study in geese, a humoral response by vaccinated birds was demonstrated by antibodies from day six onwards following live virus challenge [43]. Similar high seroconversion rates following DNA vaccination and challenge have been reported in red-tailed hawks [46] and crows [41]; however, in the hawks, seroconversion occurred in 11/14 vaccinated birds only after challenge, underlining the importance of live virus challenge in vaccine evaluation and assessment of clinical protection.

Passage of the challenge virus on Vero E6 cells may have attenuated the virus. However, virus attenuation is very unlikely, as the virus in the present study was taken as an aliquot from the same virus stock and was therefore identical to the WNV which had been used successfully in two previous infectious trials [37,51]. Moreover, similar to the non-vaccinated falcons in previous studies [37,51], the control animals in the present trial demonstrated clinical signs, viremia, virus shedding, organic virus accumulation, and pathological alterations associated to WNV infection.

There are indications that the occurrence of natural WNV infections in captive and free-ranging peregrine falcons is lower than that in gyrfalcons and gyr-hybrid-falcons, despite the opportunity for exposure. Therefore, the use of different falcon species and their hybrids might have limited the evaluation of results. However, there is no valid proof available that susceptibility to WNV varies between falcon species, as this had been stated for other viruses [62]. Moreover, the phylogenetic relationship between most of the falcon species used is so close [63-65] that differences in the immunological reaction to vaccines and virus challenge are unlikely. Accordingly, the results of the present study do not point towards any species susceptibility; however, certain host variability should be taken into account when interpreting results as it is known from other studies using non-standard laboratory animals [66].

In groups B, C, and D, clinical scores were significantly reduced and survival rates increased compared to the control group, while the survival rate of group A was low (60%). One of the two deceased falcons in group A died, most likely due to hemorrhagic enteritis, but demonstrated no signs typical for a WNV-associated disease in pathology, histopathology and IHC. It is possible that the incubation time of about 4 dpi was too short to develop clear signs of disease. A second falcon in group A and one falcon in group D had to be euthanized before the end of the trial. Interestingly, both animals demonstrated distinct amounts of antigen in the brain and/or heart by IHC, even without pathological alterations. All other vaccinated falcons (17/20) survived the challenge trial without or with mild clinical signs (*n* = 9), showing moderate to severe signs (clinical score ≥2) temporarily on single days (*n* = 4) or on 4-12 days (*n* = 4), respectively. However, in this study, the rate of survivors in groups A and D, but not in groups B and C, is lower compared to robins, where 3/3 vaccinated birds survived a challenge with WNV isolate WN02 for two weeks [45]. Similarly, 9/9 fish crows [40], 14/14 vaccinated red-tail hawks [46], and 44/44 geese [43] were protected from lethal WNV infection; however, in all of these studies, all control and mock birds also survived to the end of the infectious trial. Therefore, evaluation of the true benefits of vaccination in these studies is limited. In addition, the study of viral dissemination or pathological examinations were far less complete in those studies. This was not the case in the present study, where only 50% of the non-vaccinated control falcons survived virus challenge, suffering temporarily from moderate to severe clinical signs. Whether species-specific differences, higher virus infection doses in the present study or a different pathogenicity of the WNV challenge strains used is responsible for this remains unclear, but it highlights the fact that previous studies in other avian species are not directly comparable to the present study.

Independently of the vaccination scheme, almost all animals revealed clear histopathological signs of a mild to severe, non-suppurative (meningo-)encephalitis, which was even accompanied by a mild to moderate, non-suppurative myocarditis and/or arteritis in several falcons. Thereby, vaccination WNV-DNA-1 and WNV-DNA-2 did not prevent the development of pathological lesions; however, individual variability among the birds was high. Besides lesions which are typically associated with WNV infections, several vaccinated and non-vaccinated falcons revealed lymphohistiocytic infiltrations in different tissues. Interestingly, only in three of the surviving birds the WNV antigen was detected in brain, myocardium or spleen, suggesting an early clearance of viral antigen during the progress of disease, as discussed before [51].

In accordance with previous studies in falcons, viremia levels and virus shedding were high in non-vaccinated control falcons, especially if birds died or had to be euthanized during the trial [37]. In comparison, the levels but not the duration of oral and cloacal (except group C) virus shedding and viremia were significantly reduced in all vaccination groups. However, in the three surviving control birds, viral genome detection was also negative for single days or after 8-14 dpi, respectively. In most falcons, virus shedding extended beyond the viremic period, which has also been reported from other raptor species [46,67]. The reduction of viremia levels is in accordance with other studies. In DNA-vaccinated geese, the virus was absent from the blood; however, oral shedding occurred on 3-6 dpi, suggesting virus replication and the absence of sterile immunity [43]. In absence of mortality in the cited study, viremia during challenge has been used as a surrogate for mortality as known from different animal models for flaviviral diseases [43,68]. In robins [45] and fish-crows [40], the viremia level was decreased below the infectious level of mosquitoes, which has been reported to be 10^5^ PFU/mL blood [69]. However, this failed in crows, where viremia levels were decreased from 10^10^ only to 10^5^ PFU/mL blood [41]. In accordance to previous reports, the results of this study suggest a reduction of the transmission risk through blood sucking mosquitoes from vaccinated birds.

High viral organ loads have been reported in falcons infected with WNV lineages 1 and 2 previously [37], and correspond to findings in the deceased birds of the present study. Compared to the control group, viral load was significantly reduced in most or all organs in groups B and C and in single organs in group D, but not in group A. Similar to 2/4 surviving control birds in group E, virus genome detection was successful from the brain of all birds in group A. Moreover, in birds of group A, the viral genome was detected in kidney, heart, spleen, liver and lung of some falcons, especially in deceased animals. In contrast, the number of falcons with positive virus detection in the brain was reduced in groups B (*n* = 3) and C (*n* = 3), but not in group D (*n* = 5), and positive virus detection from other organs occurred only in one bird of group B and two birds of group D, but not in group C. This reduction of viral organ load corresponds to previous studies in falcons, where inactivated and recombinant vaccines decreased the viral organ load significantly [51].

In conclusion, the WNV DNA vaccines used in this study induced partial protection against WNV associated diseases and, depending on the route of administration, full protection from death. Interestingly, WNV-DNA-2 provided cross-protection against the WNV lineage 1 strain NY99. A similar cross-protection between different WNV lineages has been reported in horses and mouse studies [70]. Even if sterile immunity was not achieved, in many cases virus shedding, viremia and viral loads in organs were significantly decreased, reducing the risk of disease transmission from vaccinated birds. Although almost all animals developed signs of disease, the survival rate was higher compared to controls. From the practical point of view, the results of WNV-DNA-2, which was injected IM, are promising regarding the attenuation of virus shedding and viremia. Also, EP of DNA led to 100% survival of falcons; however, the set-up and availability of machines for in-vivo EP generally currently limit the extensive use of this technology for DNA-vaccines [59].
